# End-member compounds of a 4-sublattice model of multicomponent BCC solid solutions

**DOI:** 10.1016/j.dib.2018.08.086

**Published:** 2018-08-29

**Authors:** Arash Hosseinzadeh Delandar, Oleg I. Gorbatov, Malin Selleby, Yuri N. Gornostyrev, Pavel A. Korzhavyi

**Affiliations:** a Department of Materials Science and Engineering, KTH Royal Institute of Technology, SE-100 44 Stockholm, Sweden; b Applied Physics, Division of Materials Science, Department of Engineering Sciences and Mathematics, Luleå University of Technology, 97187 Luleå, Sweden; c Laboratory for Mechanics of Gradient Nanomaterials, Nosov Magnitogorsk State Technical University, 455000 Magnitogorsk, Russia; d Institute of Metal Physics, Ural Division RAS, 620219 Ekaterinburg, Russia; e Institute of Quantum Materials Science, 620107 Ekaterinburg, Russia; f National University of Science and Technology "MISiS", 119049 Moscow, Russia

## Abstract

The article presents *ab initio* calculated properties (total energies, lattice parameters, and elastic properties) for the complete set of 1540 end-member compounds within a 4-sublattice model of Fe-based solid solutions. The compounds are symmetry-distinct cases of integral site occupancy for superstructure Y (space group #227, type LiMgPdSn) chosen to represent the ordered arrangements of solvent atoms (Fe), solute atoms (Fe, Mg, Al, Si, P, S, Mn, Ni, Cu), and vacancies (Va) on the sites of a body-centered cubic lattice. The model is employed in the research article “*Ab-initio* based search for late blooming phase compositions in iron alloys” (Hosseinzadeh et al., 2018) [1].

**Specifications Table**

**Table:** 

Subject area	*Physics, Chemistry*
More specific subject area	*Computational materials science, Solid state chemistry*
Type of data	*Table, figure*
How data was acquired	*Density functional theory calculations using the VASP-PAW method*
Data format	*Raw, calculated, analyzed*
Experimental factors	*N/A*
Experimental features	*DFT calculations in the generalized gradient approximation*
Data source location	KTH Royal Institute of Technology, SE-100 44 Stockholm, Sweden
Data accessibility	*Data is provided with this article*

**Value of the data**

• The raw data for the complete set of end-member compounds, together with the compound energy model, can be used for evaluation of the properties of partially ordered multicomponent phases and for verification of the validity of other models of such phases.
• The calculated and evaluated energies allow one to identify thermodynamically favored precipitate phases in multicomponent alloys involving the elements considered.
• The calculated and evaluated lattice parameters allow one to deduce the lattice misfit of potential precipitate phases with the alloy matrix.
• The calculated cubic elastic constants can be used in the analysis of precipitation strengthening.

## 1 Data

**Fig. 1:**
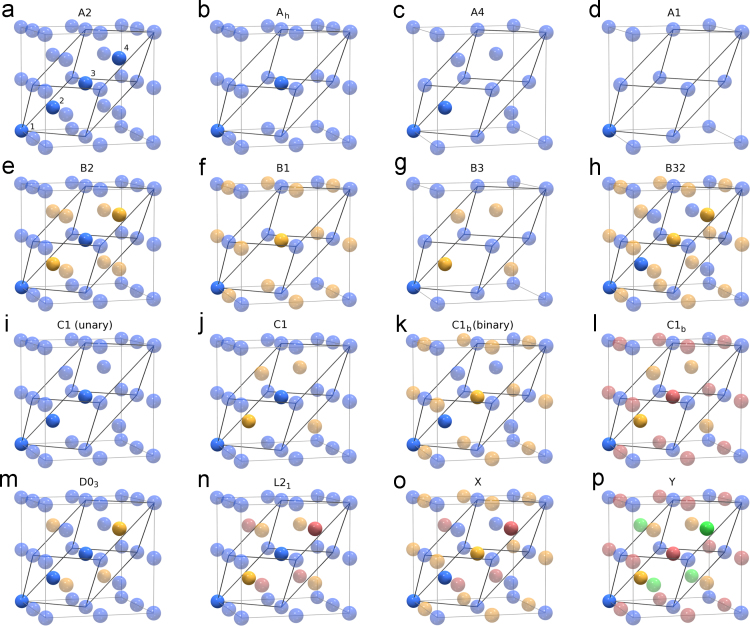
Symmetry-distinct cases of site occupancy in a face-centered cubic structure of type Y describing various ordered superstructures in multicomponent (including vacancies) solid solutions on the sites of the underlying body-centered cubic lattice. Thin gray lines show unit cell boundaries; thicker black lines indicate edges of the primitive cell. Differently colored balls denote different atomic species residing on the four sites specified in (a). The atoms belonging to the basis are shown in full color, their periodically repeated images inside the unit cell are shown as half-transparent balls. Vacancies are shown as empty sites. For each superstructure, its Strukturbericht designation is indicated above the corresponding subfigure.

The data are provided as an Excel table’Y-model.xlsx’ with *ab initio* calculated properties (raw and processed data) for 1540 end-member compounds constituting a 4-sublattice model based on the Y structure, a quaternary ordered phase (space group #227, type LiMgPdSn). A face-centered cubic unit cell of the Y structure is shown in Fig. 1p. This so-called Y-model has been used in Ref. [1] to enumerate compounds that could be obtained by ordering solute atoms (Mg, Al, Si, P, S, Mn, Ni, Cu) and vacancies (Va) on sites of the underlying body-centered cubic lattice (Fig. 1a) of Fe. The complete list of structures described by the Y-model is provided in Table 1. For each structure, Strukturbericht designation, prototype, and alternative name are given, if available. Fig. 1 depicts the atomic motif for every crystal structure described by the Y-model.

**Table 1:** Structures of end-member compounds in the 4-sublattice model of bcc solid solutions (including vacancies, indicated as’–’). The last column gives the number of different end members for a 9-component alloy.

**Occupancy**	**Designation**	**Prototype**	**Other name**	**Number of end members (9 components)**
–,–,–,–			Empty lattice	1
A,A,A,A	B2	Fe	bcc	9
A,A,A,–	C1 (unary)	CaF_2_	Unary Fluorite	9
A,A,–,–	A4	C	Diamond	9
A,–,A,–	A_*h*_	α-Po	sc	9
A,–,–,–	A1	Cu	fcc	9
A,B,A,B	B2	CsCl		36
A,A,B,B	B32	NaTl	Zintl	36
A,–,B,–	B1	NaCl	Halite	36
A,B,–,–	B3	ZnS	Sphalerite	36
A,A,A,B	D0_3_	BiF_3_		72
A,B,A,–	C1	CaF_2_	Fluorite	72
A,A,B,–	C1_*b*_ (binary)	MgAgAs	binary half-Heusler	72
A,B,C, –	C1_*b*_	MgAgAs	Half-Heusler	126
A,B,A,C	L2_1_	Cu_2_MnAl	Heusler	252
A,A,B,C	X	CuHg_2_Ti	inverse Heusler	252
A,B,C,D	Y	LiMgPdSn	Quarternary Heusler	504

The last column of Table 1 specifies, for each structure, the number of *different* end members that result from the Y-model of a 9-component solid solution on the underlying bcc crystal lattice. Obviously, the total number of end members for such an alloy, described using a 4-sublattice model and taking vacancies into account, is 10^4^. Due to the high symmetry of the Y-model many of the members are equivalent. If only the symmetry-distinct end members are counted, as it is done in the last column of Table 1, the number of *different* end members reduces to 1540, which is an order of magnitude smaller than the total number. For each of the different end member compounds (excluding the trivial case of an empty lattice) the data in the Excel table are organized as explained in Table 2.

**Table 2:** Description of data provided as Supplementary material (Excel file Y-model.xlsx).

**Column**	**Name**	**Description**	**Unit**
A	Occupancy	List of elements occupying the 4 positions in primitive unit cell, 1: (0; 0; 0), 2: (1/4; 1/4; 1/4), 3: (1/2; 1/2; 1/2), and 4: (3/4; 3/4; 3/4), see Fig. 1a.	
B	Structure	Strukturbericht designation and site occupancy pattern as in Table 1	
C	a_0	Calculated (symmetry-reduced) lattice parameter $a_{0}$ of the structure	Å
D	Delta	Calculated (linear) lattice misfit with the Fe matrix.	%
E	V	Volume per primitive cell (4 lattice sites).	Å^3^/cell
F	V/at	Volume per atom.	Å^3^/atom
G	E_tot	Calculated total energy $E_{\text{tot}}$ (per primitive cell).	eV/cell
H	E_form/at	Energy of formation ${∆E}_{\text{form}}$ calculated as Eq. (1).	eV/atom
I	E_prec/at	Energy of precipitation ${∆E}_{\text{prec}}$ calculated as Eq. (2).	eV/atom
J	C_11	Cubic elastic constant $C_{11}$.	kbar
K	C_12	Cubic elastic constant $C_{12}$.	kbar
L	C_44	Cubic elastic constant $C_{44}$.	kbar
M	C’	Tetragonal shear modulus $C^{\prime }=(C_{11}-C_{12})/2$.	kbar
N	B	Bulk modulus $B=(C_{11}+{2C}_{12})/3$.	kbar

## 2 Methods

For each of the considered structures, the total energy $E_{\text{tot}}$ and equilibrium lattice parameter $a_{0}$ were computed using spin-polarized PAW-VASP electronic structure calculations [2], [3]. The convergence criteria were 10^−6^ eV/atom for the total energy and 10^−3^ eV/Å for the forces. The PAW-VASP calculations were performed in the generalized gradient approximation [4] using a kinetic energy cutoff of 350 eV and a uniform 12 × 12 × 12 meshes of **k**-points determined according to the Monkhorst-Pack scheme [5]. The first-order Methfessel-Paxton smearing scheme [6] with a smearing parameter σ=0.2 eV was used for Brillouin zone integration. The mean-square deviation of the so obtained total energies from the respective total energies evaluated using the improved tetrahedron method [7] on the same **k**-mesh is found to be 1.6 meV.

As a standard indicator of phase stability, the energy of formation ${∆E}_{\text{form}}$ has been calculated for each end member, from the corresponding total energy $E_{\text{tot}}$ obtained as described above and expressed relative to the total energies $E_{\text{ref}}$ (per atom) of the elements in the following reference states: bcc Fe (ferromagnetic), fcc Ni (ferromagnetic), Cu, Mg, and Al, diamond Si, and simple cubic (sc) P and S. The reference state structure for Mn was generated by imposing the AFM-I antiferromagnetic order in the fcc Mn and fully relaxing the obtained tetragonal structure. Note that for Mg, P, S, and Mn elements, the reference state structure was chosen to be different from their respective ground state structures at T=0, for the sake of simplicity. The reference energy for Va was naturally taken to be zero. The formation energy of a general end member ׳ABCD׳ compound was then expressed per atom, not counting vacancies as real atoms, as follows:

(1) $${∆E}_{\text{form}}\left(\text{ABCD}\right)=\left[E_{\text{tot}}\left(\text{ABCD}\right)-\sum_{X=\text{A,B,C,D}}E_{\text{ref}}\left(X\right)\right]/(4-N_{\text{Va}}).$$

where $N_{\text{Va}}$ is the number of vacant sites among the 4 positions in the structure basis, see Table 1.

To have a complementary indicator of stability of precipitate phases, we also evaluate the thermodynamic driving force for precipitation ${∆E}_{\text{form}}$ (also referred to as energy of precipitation) by replacing the reference state energies in Eq. (1) with chemical potentials $\mu_{X}^{0}$ of the elements (but not vacancies) in the dilute Fe-based solid solution as:

(2) $${∆E}_{\text{prec}}\left(\text{ABCD}\right)=\left[E_{\text{tot}}\left(\text{ABCD}\right)-\sum_{X=\text{A,B,C,D}}\mu_{X}^{0}\right]/(4-N_{\text{Va}}).$$

The chemical potentials (equivalent to solution energies in the T→0 limit) for individual solutes or vacancies in the bcc Fe matrix, the isolated defects were considered in a 108-site supercell obtained by triplication of the 4-atom primitive unit cell of the Y-model along its primitive translation vectors (3×3×3×4). The chemical potential for a substitutional solute element *X* was calculated as follows:

(3) $$\mu_{X}^{0}=E_{\text{tot}}\left({\text{Fe}}_{107}X\right)-\frac{107}{108}E_{\text{tot}}\left({\text{Fe}}_{108}\right).$$

Finally, the set of cubic elastic constants was determined for every end-member compound of the Y-model at the respective calculated equilibrium volume by applying symmetry-required lattice distortions, with four strain values at a step of 0.01, and following the stress-strain relationships as described in Ref. [8].
